# Integrating Gene Expression and Protein Interaction Data for Signaling Pathway Prediction of Alzheimer's Disease

**DOI:** 10.1155/2014/340758

**Published:** 2014-04-09

**Authors:** Wei Kong, Jingmao Zhang, Xiaoyang Mou, Yang Yang

**Affiliations:** ^1^Information Engineering College, Shanghai Maritime University, Shanghai 201306, China; ^2^DNJ Pharma and Rowan University, Glassboro, NJ 08028, USA; ^3^Department of Computer Science and Engineering, Shanghai Jiao Tong University, Shanghai 200240, China

## Abstract

Discovering the signaling pathway and regulatory network would provide significant advance in genome-wide understanding of pathogenesis of human diseases. Despite the rich transcriptome data, the limitation for microarray data is unable to detect changes beyond transcriptional level and insufficient in reconstructing pathways and regulatory networks. In our study, protein-protein interaction (PPI) data is introduced to add molecular biological information for predicting signaling pathway of Alzheimer's disease (AD). Combining PPI with gene expression data, significant genes are selected by modified linear regression model firstly. Then, according to the biological researches that inflammation reaction plays an important role in the generation and deterioration of AD, NF-**κ**B (nuclear factor-kappa B), as a significant inflammatory factor, has been selected as the beginning gene of the predicting signaling pathway. Based on that, integer linear programming (ILP) model is proposed to reconstruct the signaling pathway between NF-**κ**B and AD virulence gene APP (amyloid precursor protein). The results identify 6 AD virulence genes included in the predicted inflammatory signaling pathway, and a large amount of molecular biological analysis shows the great understanding of the underlying biological process of AD.

## 1. Introduction

Alzheimer's disease (AD) is a progressive and fatal neurodegenerative disorder manifested by cognitive and memory deterioration. The characteristic pathology changes in AD are fibrin deposition in cerebral cortex; it is the deposition of beta-amyloid (A*β*) in cell space and poly-Tau protein in cell. In pathomorphism, the expression is senile plaques (SP) and neurofibrillary tangles (NFT).

Many studies have investigated the mechanism of AD from various perspectives of its complexity. Recent researches show that a more accepted hallmark of AD is brain inflammation. Inflammation clearly occurs in pathologically vulnerable regions of AD brain and it does so with the full complexity of local peripheral inflammatory responses [1–3]. In the periphery, degenerating tissue and the deposition of highly insoluble abnormal materials are classical stimulants of inflammation. Likewise, in the AD brain damaged neurons and neurites and highly insoluble A*β* peptide deposits and neurofibrillary tangles provide obvious stimuli for inflammation [4–7].

To give insight to the AD mechanisms, high-throughput gene expression data has received extensive attention and made substantial progress in reconstructing the gene regulatory network. However, due to the underlying shortcomings of microarray technology such as small sample size, measurement error, and information insufficiency, unveiling disease mechanism has remained a major challenge to the AD research community. To overcome these problems, pathway information and network-based approaches [8] have been applied and become more informative and powerful for discovering disease mechanism.

Protein-protein interaction (PPI) networks are reconstructed from protein domain characteristics, gene expression data, and structure-based information with other evidence, for example, gene homology, function annotations, and sequence motifs [9]. PPI data contain structure information among different genes while gene expression data do not. In our study, PPI network data as a priori pathway information is introduced for predicting the inflammatory signaling pathway in AD. Many literatures have given outstanding achievements by integrating gene expression data and PPI data, such as identification of protein complexes [10], small subnetworks [11], and biomarkers [12]. Zhao et al. presented an integer linear programming (ILP) method to uncover pathways among the given starting proteins, ending proteins, and some transduction factor proteins [13]. However, how to select the transduction factor proteins is a great problem. In our study, a modified network-constrained regularization analysis method [14] is proposed for linear regression analysis to select appropriate number of significant genes. Simulation results show that this method can lead to an efficiently global smoothness of regression coefficients.

Based on that, ILP model is presented to reconstruct the inflammatory signaling pathway by integrating PPI data with the AD gene expression data. In the ILP model, the starting and ending proteins of the predicting pathway need to be arranged in advance. Nuclear transcription factor NF-*κ*B (nuclear factor-kappa B) as one of the most important inflammatory factors is selected as the starting gene of the signaling pathway. As we know that NF-*κ*B plays a key role in regulating the immune response to infection, therefore incorrect regulation of NF-*κ*B has been linked to cancer, inflammatory and autoimmune diseases, septic shock, viral infection, and improper immune development. NF-*κ*B has also been implicated in processes of synaptic plasticity and memory [15]. On the other hand, APP (amyloid precursor protein) as the most important AD virulence gene and precursor protein of A*β* is arranged as the ending protein of the predicting pathway.

The experiment results show that 6 AD virulence genes are identified being included in the predicted inflammatory signaling pathway, and a large amount of inflammation related genes and pathways has been found by molecular biological analysis and they show the great understanding of the pathogenesis of AD.

## 2. Methods

### 2.1. Linear Regression Model

Linear regression model is widely used in estimation and variable selection. In our study, the model is applied to selected subset of significant genes which are important for AD and are going to be the transduction factors of reconstructing pathway. In the next prediction step, gene expression data and PPI data will be integrated by ILP model. After all of the above, a pathway could be identified between NF-*κ*B and APP. The usual linear regression model can be expressed as

(1) $$\begin{array}{c} \mu =\overset{p}{\underset{j=1}{\sum }}x_{j}\beta_{j}=x_{1}\beta_{1}+x_{2}\beta_{2}+\cdots +x_{p}\beta_{p}, \end{array}$$

where *μ* = (*μ*_1_, *μ*_2_,…,*μ*_*n*_)^*T*^ is response vector, *n* is the sample number, *x*_*j*_ = (*x*_1_, *x*_2_,…,*x*_*n*_)^*T*^, *j* = 1,2, …, *p*, is the predictor, represented by the *j*th gene's expression data in all samples, and *β*_*j*_ is the *j*th gene's weight vector. Assume that the predictors are standardized and the response is centered, we get

(2) ∑i=1n μi=0,  ∑i=1nxij=0,  ∑i=1nxij2=0,for  j=1,2,…,p.

Gene expression data has the characteristic of less sample and great noise. As a simple model, linear regression model has significant performance in handling less sample and great noise data. The significant genes will get a larger coefficient while the nonsignificant genes will get a smaller coefficient.

### 2.2. Network-Constrained Regularization for the Linear Regression Model

Before using linear regression model, coefficient *β* needs to be estimated. Many methods have been proposed which focused on addressing high-dimensionality genomic data such as LASSO, LA-SEN, and LARS. Here, a modified network-constrain regularization analysis by C. Li and H. Li [14] is applied to estimate the coefficient since it has been proved to perform better than other methods. This method is a lasso-type problem. It defines a normalized Laplacian matrix *L* as

(3) $$\begin{array}{c} L=\{\begin{array}{cc} \frac{1-w\left(u,v\right)}{d_{u}} & \text{if}\;u=v\;\text{and}\;d_{u}\neq 0,\\ \frac{-w\left(u,v\right)}{\sqrt{d_{u}d_{v}}} & \text{if}\;u\;\text{and}\;v\;\text{are}\;\text{adjacent},\\ 0 & \text{otherwise}, \end{array} \end{array}$$

where *w*(*u*, *v*) represents the weight of edge between linked genes *u* and *v*. *d*_*v*_ = ∑_*u*~*v*_ *w*(*u*, *v*) represents all the adjacent genes of *v* on the network. Then the definition of the network-constrained regularization criterion is

(4) $$\begin{array}{c} L\left(\lambda_{1},\lambda_{2},\beta \right)={\left(\mu -X\beta \right)}^{T}\left(\mu -X\beta \right)+\lambda_{1}{\left|\beta \right|}_{1}+\lambda_{2}\beta^{T}L\beta , \end{array}$$

where *X* = (*x*_1_|*K*|*x*_*p*_); |*β*_1_| = ∑_*j*=1_^*p*^|*β*_*j*_|; *λ*_1_, *λ*_2_ are nonnegative turning parameters. And then we estimate *β* by minimizing (4):

(5) $$\begin{array}{c} \beta =\underset{\beta }{\text{argmin}}\;\left\{L\left(\lambda_{1},\lambda_{2},\beta \right)\right\}. \end{array}$$

Minimizing (4) is equivalent to solving a lasso-type optimization problem. Turning parameters are estimated by 10-fold cross-validation (CV). Genes in gene interaction network are selected by PubGene; we chose genes related to Alzheimer.

### 2.3. Integer Linear Programming (ILP)

The ILP model formulates signaling network detection as an optimization problem and treats a signaling network as a whole entity as described in its original publication [13]. PPI network is a weighted undirected graph, that can be described as **G**(**V**, **E**, **W**), where **V** is vertices in the graph, representing protein; **E** is edge between proteins; and **W** represents the weight of edges. **W** can be calculated by gene expression data. The ILP model can be described as follows:

(6) Minmize{xi,yij}⁡   S=−∑i=1|V|∑j=1|V|wijyij+λ∑i=1|V|∑j=1|V|yijSubject  to: yij≤xi,yij≤xj,∑j=1|V|yij≥1, if  i  is  either  a  startingor  ending  protein,∑j=1|V|yij≥2xi, if  i  is  not  a  starting or  ending  protein,xi=1, if  i  is  a  protein  known  in  STN,xi∈{0,1}, i=1,2,…,|V|,yij∈{0,1}, i,j=1,2,…,|V|,

where *w*_*ij*_ is the weight between proteins *i* and *j* in weighted undirected graph *G*; *y*_*ij*_ is a binary variable to denote whether the edge *E*(*i*, *j*) is a part of the STN. *x*_*i*_ is also a binary variable to denote whether protein *i* is a component of the STN. *λ* is a positive penalty parameter. |*V*| includes all proteins in the PPI network. *y*_*ij*_ ≤ *x*_*i*_ and *y*_*ij*_ ≤ *x*_*j*_ mean that only if proteins *i* and *j* are both components of STN, the edge *E*(*i*, *j*) should be considered. ∑_*j*=1_^|*V*|^ *y*_*ij*_ ≥ 1 represents at least one protein contact with starting protein or ending protein. ∑_*j*=1_^|*V*|^ *y*_*ij*_ ≥ 2*x*_*i*_ makes sure that if *x*_*i*_ is selected as a component of STN, there are at least two proteins link to the vertex.

The starting protein and ending protein have confirmed above that the genes selected by linear model were treated as transduction factors. The method of chosen parameter *λ* can be found in its original publication. Then we detected protein pathway by the ILP model.

## 3. Results and Discussion

To evaluate ILP model, AD dataset, series GSE1297, was used which were human hippocampal gene expression downloaded from GEO DataSets from the National Center for Biotechnology Information (NCBI) offered by Blalock et al. [16]. The hippocampal specimens they used are obtained through the Brain Bank of the Alzheimer's Disease Research Center at the University of Kentucky. The human Gene Chips (HG-U133A) of Affymetrix and Microarray Suite 5 are used in analyzing the microarray data. There are a total of 9 control, 7 incipient, 8 moderate, and 7 severe AD samples included in this dataset with 22283 gene expressions in each sample. The PPI data we used is downloaded from website BioGRID (http://thebiogrid.org/) with 12466 proteins and 40323 interactions in total.

**Figure 1:**
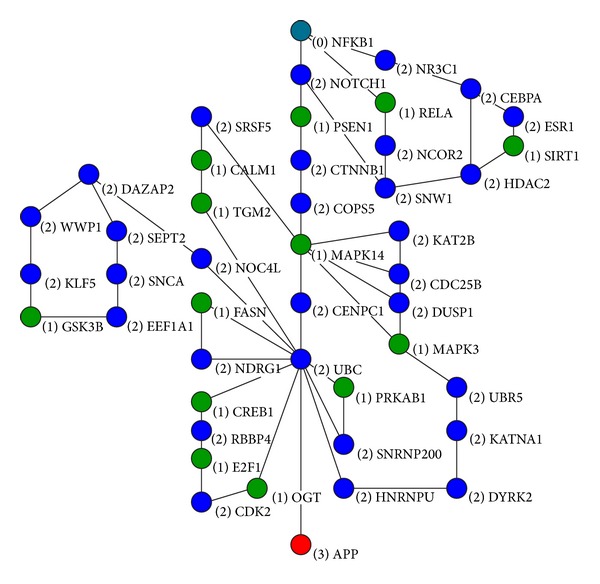
Protein signaling pathway predicting result between NF-*κ*B and APP.

The file format of microarray data downloaded from NCBI is CEL. The probe data needs data processing like background correct, normalization, probe correct, and so on. Then ANOVAs were used on preliminary select genes and removed all genes whose *P* value was less than 0.05. After processing, 7030 genes for each sample were left. Then taking linear regression model with modified network-constrained regularization and AD biological information, the coefficients *β* = (*β*_1_, *β*_2_,…, *β*_*p*_)  and  *p* = 7030 were obtained. Among them, 7017 values of *β* were zeroes and the other 13 *βs*were nonzero values. So these 13 genes with nonzero values of *β*  were considered as significant genes to AD phenotype and they are denoted in green circles with “(1)” and gene names in Figure 1.

The 13 selected genes can be mapped to PPI network to get the corresponding proteins and the interactions between them and other proteins. Each selected gene was connected with some other genes in PPI network by the edges. In ILP algorithm, edge between *i* and *j* was represented by *y*_*ij*_. When ILP chose this edge, *y*_*ij*_ = 1, otherwise *y*_*ij*_ = 0.

ILP tries to assign 0 or 1 for *y*_*ij*_ to ensure the result network has the largest weight. For the weight of edge, *w*_*ij*_, here we use the Pearson coefficient of the gene expression values to represent the weight between proteins *i* and *j*.

Then using NF-*κ*B as starting protein and APP as ending protein ILP model was applied to formulate the signaling network. In the ILP algorithm, penalty parameter *λ* is a size control parameter that needs to be adapted manually. If its value is too large, the predicted signaling network will be enormous, otherwise it will be too small to catch the useful biological information. In our simulation experiment, after adapting from small value to large value, *λ* was determined as 0.65.

We finally got a signaling pathway with several small subnetworks. This network is reconstructed by 45 genes including 13 selected significant genes and is shown in Figure 1.

In Figure 1, “(0) NF-*κ*B” represents the starting protein NF-*κ*B, “(3) APP” represents the ending protein APP, “(1)” with the protein names denote the corresponding selected genes by the regress model, and “(2)” with protein names are selected by ILP to reconstruct the signaling pathways between NF-*κ*B and APP. In order to analyze the biological functions of the pathways and subnetworks, the predicted result was mapped into its coding gene pathway network, and the online analysis website DAVID (http://david.abcc.ncifcrf.gov/home.jsp) was utilized to further understand their molecular biological functions to AD.  Table 1 shows the KEGG pathway analysis result.

First of all, among the prediction results, there are 5 genes that have been confirmed as the AD virulence genes such as SNCA, CALM1, GSK3B, PSEN1, and APP which have been biologically demonstrated playing crucial roles in AD. Based on Table 1, T cell receptor signaling pathway, B cell receptor signaling pathway, the Notch signaling pathway, NOD-like receptor signaling pathway, Toll-like receptor signaling pathway, MAPK signaling pathway, neurotrophin signaling pathway, insulin signaling pathway, and so on were found to include a major part of important genes derived from the regression model. Specially, the main predicted pathway in Figure 1 includes NFKB1, NOTCH1, PSEN1, CTNNB, COPS5, MAPK14, CENPC1, UBC, and APP; the molecular biological analysis shows that they have close correlation between inflammatory response and AD.

It was found that inflammation is a major mechanism of acute brain injury and chronic neurodegeneration [17]. During the onset of an inflammatory response, signaling pathways are activated for translating extracellular signals into intracellular responses converging to the activation of NF-*κ*B, the central transcription factor in driving the inflammatory response [18]. NF-*κ*B has long been considered a prototypical proinflammatory signaling pathway, largely based on the activation of NF-*κ*B by proinflammatory cytokines, such as interleukin-1 (IL-1) and tumor necrosis factor *α* (TNF*α*), and the role of NF-*κ*B on the expression of other proinflammatory genes including cytokines, chemokines, and adhesion molecules, which has been extensively reviewed elsewhere [9].

Recent studies have also found that Notch receptors in Notch signaling pathway regulate cell differentiation and function, and Notch1 has been shown to induce glia in the peripheral nervous system [19, 20]. NF-*κ*B, Notch, MAKP, and PSEN1 included in the main pathway of Figure 1 were observed to have strong regulating functions between each other, since interleukin-1 (IL-1) activates NF-*κ*B via interleukin-1 receptor-associated kinase (IRAK) and mitogen-activated protein kinase (MEKK1(MAP3 K)) dependent inhibition of NF-*κ*B inhibitor (I-*κ*B) [21, 22]. V-rel reticuloendotheliosis viral oncogene homolog (c-Rel (NF-*κ*B subunit)) can trigger Notch homolog 1 translocation-associated (NOTCH1 receptor) signaling pathway by inducing expression of Jagged1, ligand for Notch receptors [23, 24]. NOTCH1 receptor activated by Jagged1 or Delta-like 1 (DLL1) is cleaved by ADAM metallopeptidase domain 17 (ADAM17) and PSEN1 to intracellular domain of NOTCH1. NOTCH1 is transported to nucleus and participates in recombination signal binding protein for immunoglobulin kappa J region (RBP-J kappa (CBF1)) mediated transcription [24, 25].

It was also found that  *β*-catenin- (CTNNB1-) dependent WNT signaling pathways have crucial roles in the regulation of diverse cell behaviours, including cell fate, proliferation, survival, differentiation, migration, and polarity [26, 27]. It is interesting to note that loss of TNF*α* function would inhibit Wnt/*β*-catenin signaling [28]. Recently studies show that Wnt/*β*-catenin and NF-*κ*B are independent pathways; cross-regulation between the Wnt and NF-*κ*B signaling pathways has emerged as an important area for the regulation of a diverse array of genes and pathways active in chronic inflammation, immunity, development, and tumorigenesis. Both *β*-catenin and NF-*κ*B activate inducible nitric oxide synthase (iNOS) gene expression [29].

In addition, the regulatory network between COPS5 and CENPC1 has been extracted from our algorithm which is also observed to be implicated in the pathogenesis of AD. The COP9 (constitutive photomorphogenesis 9) signalosome (COPS), a large multiprotein complex that resembles the 19S lid of the 26S proteasome, plays a central role in the regulation of the E3-cullin RING ubiquitin ligases (CRLs). The catalytic activity of the COPS complex, carried by subunit 5 (COPS 5/Jab1), COPS-dependent COPS 5, displays isopeptidase activity; it is intrinsically inactive in other physiologically relevant forms [30]. Increased APP and accumulation of neurotoxic A*β* in the brain are central to the pathogenesis of AD. COPS5 is found to be a novel RanBP9-binding protein that increases APP processing and A*β* generation [31]. COPS5 regulates the stability of the inner kinetochore components CENP-T and CENP-W, providing the first direct link between COPS5 and the mitotic apparatus and highlighting the role of COPS5 as a multifunctional cell cycle regulator [32]. CENP-T interacts with both centromeric chromatin and microtubule binding kinetochore complexes. Transient targeting of CENP-C to a noncentromere LacO locus induces the recruitment of some outer kinetochore proteins, similar to CENP-T [33]. Our result exhibited that COPS5 regulates CENP-C in the main pathway and ubiquitin and NF-*κ*B were found to be associated with them. Ubiquitin can degrade the I*κ*B which is the inhibitor of NF-*κ*B, processing of precursors, and activation of the I*κ*B kinase (IKK) through a degradation-independent mechanism [34]. On the other hand, COPS5 functions through CDK2 to control premature senescence in a novel way, depending on cyclin E in the cytoplasm [35].

## 4. Conclusions

Although many efforts have been done several decades of AD, it is still difficult to uncover its phenotype-pathway relationship and pathogenesis. Recent studies show that the pathology of AD has an inflammatory component that is characterized by upregulation of proinflammatory cytokines, particularly in response to A*β*. However, the signaling pathways and regulatory networks of the inflammation in AD pathogenesis are very difficult to reconstruct due to the complexity.

To discover the inflammation signaling pathway and regulatory network of AD, in our study, protein interactive network data, PPI was introduced to overcome the information insufficiencies of DNA microarray gene expression data by integer linear programming (ILP) method. Two stages had been used in predicting inflammatory pathway for AD. Firstly, significant genes had been selected by linear regression analysis with the modified network-constrained regularization analysis. Then ILP model was applied to reconstruct the signaling pathway between NF-*κ*B and AD virulence gene APP since NF-*κ*B has long been considered a prototypical proinflammatory signaling pathway. From the molecular biology analysis, we found that genes on the main pathway of the reconstruction results play crucial roles in inflammatory response and APP which give more biological insight for AD pathogenesis, such as NF-*κ*B, NOTCH1, CTNNB1, COPS5, and their signaling pathways. Even more, the pathogenic contribution of the inflammatory response in AD is supported by our finding of the regulating and functions of the genes and subnetworks in the predicted signaling pathways. In general, our studies on combining PPI and gene expression data discover the signaling pathways of inflammatory response on AD and help for deeply understanding the pathogenesis of AD.

## Figures and Tables

**Table 1:** KEGG pathway analysis of the predicted pathways and subnetworks in Figure 1.

Pathway	Number of genes
Prostate cancer	9
Notch signaling pathway	8
Neurotrophin signaling pathway	6
Pathways in cancer	6
Chronic myeloid leukemia	6
Alzheimer's disease	5
Melanogenesis	9
T cell receptor signaling pathway	5
Acute myeloid leukemia	5
NOD-like receptor signaling pathway	5
Cell cycle	5
Insulin signaling pathway	6
Pancreatic cancer	4
B cell receptor signaling pathway	4
Small cell lung cancer	4
Progesterone-mediated oocyte maturation	4
MAPK signaling pathway	4
Toll-like receptor signaling pathway	5
Endometrial cancer	6
Spliceosome	4
Glioma	5
Adipocytokine signaling pathway	3
Epithelial cell signaling in *Helicobacter pylori* infection	3
RIG-I-like receptor signaling pathway	3
Colorectal cancer	3

## References

[1] Haruhiko A, Barger S, Barnum S (2000). Inflammation and Alzheimer's disease. *Neurobiology of Aging*.

[2] Meyer-Luehmann M, Spires-Jones TL, Prada C (2008). Rapid appearance and local toxicity of amyloid-*β* plaques in a mouse model of Alzheimer’s disease. *Nature*.

[3] Lee Y-J, Han SB, Nam S-Y, Oh K-W, Hong JT (2010). Inflammation and Alzheimer’s disease. *Archives of Pharmacal Research*.

[4] Galimberti D, Scarpini E (2011). Inflammation and oxidative damage in Alzheimer’s disease: friend or foe?. *Frontiers in Bioscience*.

[5] Johnston H, Boutin H, Allan SM (2011). Assessing the contribution of inflammation in models of Alzheimer’s disease. *Biochemical Society Transactions*.

[6] Holmes C, Butchart J (2011). Systemic inflammation and Alzheimer’s disease. *Biochemical Society Transactions*.

[7] Lan X, Liu R, Sun L, Zhang T, Du G (2011). Methyl salicylate 2-O-*β*-D-lactoside, a novel salicylic acid analogue, acts as an anti-inflammatory agent on microglia and astrocytes. *Journal of Neuroinflammation*.

[8] Bhardwaj N, Lu H (2005). Correlation between gene expression profiles and protein-protein interactions within and across genomes. *Bioinformatics*.

[9] Huang Y, Sun X, Hu G (2011). An integrated genetics approach for identifying protein signal pathways of Alzheimer’s disease. *Computer Methods in Biomechanics and Biomedical Engineering*.

[10] Feng J, Jiang R, Jiang T (2011). A max-flow-based approach to the identification of protein complexes using protein interaction and microarray data. *IEEE/ACM Transactions on Computational Biology and Bioinformatics*.

[11] Huang Y, Zhang J, Huang Y Computational identification of proteins sub-network in Parkinson's disease study.

[12] Jahid MJ, Ruan J Identification of biomarkers in breast cancer metastasis by integrating protein-protein interaction network and gene expression data.

[13] Zhao X-M, Wang R-S, Chen L, Aihara K (2008). Uncovering signal transduction networks from high-throughput data by integer linear programming. *Nucleic Acids Research*.

[14] Li C, Li H (2008). Network-constrained regularization and variable selection for analysis of genomic data. *Bioinformatics*.

[15] Zhou X, Yuan L, Zhao X (2014). Genistein antagonizes inflammatory damage induced by *β*-amyloid peptide in microglia through TLR4 and NF-*κ*B. *Nutrition*.

[16] Blalock EM, Geddes JW, Chen KC, Porter NM, Markesbery WR, Landfield PW (2004). Incipient Alzheimer’s disease: microarray correlation analyses reveal major transcriptional and tumor suppressor responses. *Proceedings of the National Academy of Sciences of the United States of America*.

[17] Rolova T, Puli L, Magga J (2013). Complex regulation of acute and chronic neuroinflammatory responses in mouse models deficient for nuclear factor kappa B p50 subunit. *Neurobiology of Disease*.

[18] Foteinou PT, Calvano SE, Lowry SF, Androulakis IP (2009). In silico simulation of corticosteroids effect on an NFkB-dependent physicochemical model of systemic inflammation. *PLoS ONE*.

[19] Zanotti S, Ernesto C (2014). *Notch1* and *Notch2* expression in osteoblast precursors regulates femoral microarchitecture. *Bone*.

[20] Tanigaki K, Nogaki F, Takahashi J, Tashiro K, Kurooka H, Honjo T (2001). *Notch1* and *Notch3* instructively restrict bFGF-responsive multipotent neural progenitor cells to an astroglial fate. *Neuron*.

[21] Renard P, Raes M (1999). The proinflammatory transcription factor NF*κ*B: a potential target for novel therapeutical strategies. *Cell Biology and Toxicology*.

[22] Yao J, Tae WK, Qin J (2007). Interleukin-1 (IL-1)-induced TAK1-dependent Versus MEKK3-dependent NF*κ*B activation pathways bifurcate at IL-1 receptor-associated kinase modification. *The Journal of Biological Chemistry*.

[23] Bash J, Zong W-X, Banga S (1999). Rel/NF-*κ*B can trigger the Notch signaling pathway by inducing the expression of Jagged1, a ligand for Notch receptors. *The EMBO Journal*.

[24] Osipo C, Golde TE, Osborne BA, Miele LA (2008). Off the beaten pathway: the complex cross talk between Notch and NF-*κ*B. *Laboratory Investigation*.

[25] Bolós V, Grego-Bessa J, De La Pompa JL (2007). Notch signaling in development and cancer. *Endocrine Reviews*.

[26] Anastas JN, Moon RT (2013). WNT signalling pathways as therapeutic targets in cancer. *Nature Reviews Cancer*.

[27] Nejak-Bowen KN, Monga SPS (2011). Beta-catenin signaling, liver regeneration and hepatocellular cancer: sorting the good from the bad. *Seminars in Cancer Biology*.

[28] Gong M, Liu C, Zhang L (2014). Loss of the TNF*α* function inhibits Wnt/*β*-catenin signaling, exacerbates obesity development in adolescent spontaneous obese mice. *Molecular and Cellular Biochemistry*.

[29] Du Q, Geller DA (2010). Cross-regulation between Wnt and NF-*κ*B signaling pathways. *Forum on Immunopathological Disease Therapeutics*.

[30] Echalier A, Pan Y, Birol M (2013). Insights into the regulation of the human COP9 signalosome catalytic subunit, CSN5/Jab1. *, Proceeding of National Academy of Science of USA*.

[31] Wang H, Dey D, Carrera I (2013). COPS5 (Jab1) protein increases *β* site processing of amyloid precursor protein and amyloid *β* peptide generation by stabilizing RanBP9 protein levels. *The Journal of Biological Chemistry*.

[32] Chun Y, Lee M, Park B (2013). CSN5/JAB1 interacts with the centromeric components CENP-T and CENP-W and regulates their proteasome-mediated degradation. *The Journal of Biological Chemistry*.

[33] Gascoigne KE, Takeuchi K, Suzuki A, Hori T, Fukagawa T, Cheeseman IM (2011). Induced ectopic kinetochore assembly bypasses the requirement for CENP-A nucleosomes. *Cell*.

[34] Chen ZJ (2005). Ubiquitin signalling in the NF-*κ*B pathway. *Nature Cell Biology*.

[35] Yoshida A, Yoneda-Kato N, Kato JY (2013). CSN5 specifically interacts with CDK2 and controls senescence in a cytoplasmic cyclin E-mediated manner. *Science Reports*.

